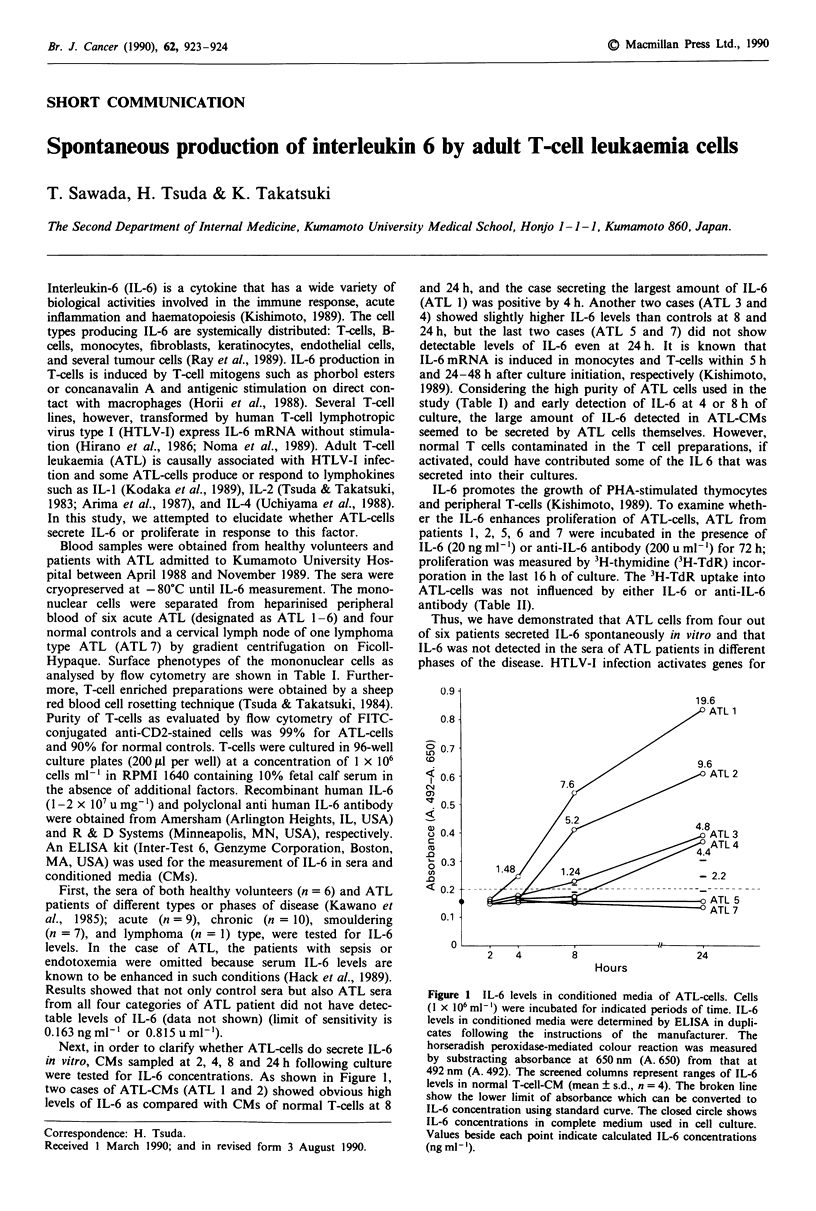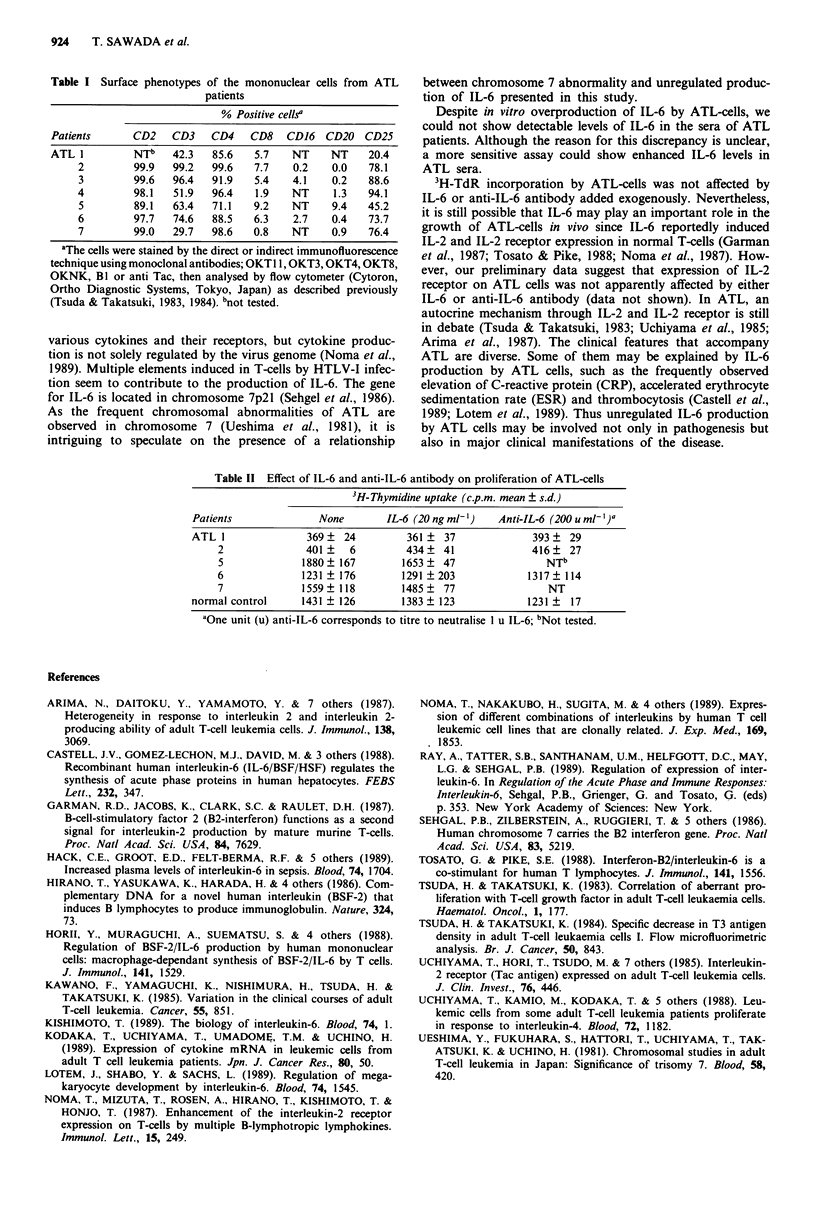# Spontaneous production of interleukin 6 by adult T-cell leukaemia cells.

**DOI:** 10.1038/bjc.1990.410

**Published:** 1990-12

**Authors:** T. Sawada, H. Tsuda, K. Takatsuki

**Affiliations:** Second Department of Internal Medicine, Kumamoto University Medical School, Japan.


Br. J. Cancer (1990), 62, 923-924 © Macmillan Press Ltd., 1990

SHORT COMMUNICATION

### Spontaneous production of interleukin 6 by adult T-cell leukaemia cells

T. Sawada, H. Tsuda & K. Takatsuki

*The Second Department of Internal Medicine, Kumamoto University Medical School, Honjo 1-1-1, Kumamoto 860, Japan.*

Interleukin-6 (IL-6) is a cytokine that has a wide variety of biological activities involved in the immune response, acute inflammation and haematopoiesis (Kishimoto, 1989). The cell types producing IL-6 are systemically distributed: T-cells, B-cells, monocytes, fibroblasts, keratinocytes, endothelial cells, and several tumour cells (Ray *et al.*, 1989). IL-6 production in T-cells is induced by T-cell mitogens such as phorbol esters or concanavalin A and antigenic stimulation on direct contact with macrophages (Horii *et al.*, 1988). Several T-cell lines, however, transformed by human T-cell lymphotropic virus type I (HTLV-I) express IL-6 mRNA without stimulation (Hirano *et al.*, 1986; Noma *et al.*, 1989). Adult T-cell leukaemia (ATL) is causally associated with HTLV-I infection and some ATL-cells produce or respond to lymphokines such as IL-1 (Kodaka *et al.*, 1989), IL-2 (Tsuda & Takatsuki, 1983; Arima *et al.*, 1987), and IL-4 (Uchiyama *et al.*, 1988). In this study, we attempted to elucidate whether ATL-cells secrete IL-6 or proliferate in response to this factor.

Blood samples were obtained from healthy volunteers and patients with ATL admitted to Kumamoto University Hospital between April 1988 and November 1989. The sera were cryopreserved at $-80°C$ until IL-6 measurement. The mononuclear cells were separated from heparinised peripheral blood of six acute ATL (designated as ATL 1-6) and four normal controls and a cervical lymph node of one lymphoma type ATL (ATL 7) by gradient centrifugation on Ficoll-Hypaque. Surface phenotypes of the mononuclear cells as analysed by flow cytometry are shown in Table I. Furthermore, T-cell enriched preparations were obtained by a sheep red blood cell rosetting technique (Tsuda & Takatsuki, 1984). Purity of T-cells as evaluated by flow cytometry of FITC-conjugated anti-CD2-stained cells was 99% for ATL-cells and 90% for normal controls. T-cells were cultured in 96-well culture plates (200 µl per well) at a concentration of $1 \times 10^6$ cells $ml^{-1}$ in RPMI 1640 containing 10% fetal calf serum in the absence of additional factors. Recombinant human IL-6 ($1-2 \times 10^7$ u $mg^{-1}$) and polyclonal anti human IL-6 antibody were obtained from Amersham (Arlington Heights, IL, USA) and R & D Systems (Minneapolis, MN, USA), respectively. An ELISA kit (Inter-Test 6, Genzyme Corporation, Boston, MA, USA) was used for the measurement of IL-6 in sera and conditioned media (CMs).

First, the sera of both healthy volunteers ($n = 6$) and ATL patients of different types or phases of disease (Kawano *et al.*, 1985); acute ($n = 9$), chronic ($n = 10$), smouldering ($n = 7$), and lymphoma ($n = 1$) type, were tested for IL-6 levels. In the case of ATL, the patients with sepsis or endotoxemia were omitted because serum IL-6 levels are known to be enhanced in such conditions (Hack *et al.*, 1989). Results showed that not only control sera but also ATL sera from all four categories of ATL patient did not have detectable levels of IL-6 (data not shown) (limit of sensitivity is 0.163 ng $ml^{-1}$ or 0.815 u $ml^{-1}$).

Next, in order to clarify whether ATL-cells do secrete IL-6 *in vitro*, CMs sampled at 2, 4, 8 and 24 h following culture were tested for IL-6 concentrations. As shown in Figure 1, two cases of ATL-CMs (ATL 1 and 2) showed obvious high levels of IL-6 as compared with CMs of normal T-cells at 8 and 24 h, and the case secreting the largest amount of IL-6 (ATL 1) was positive by 4 h. Another two cases (ATL 3 and 4) showed slightly higher IL-6 levels than controls at 8 and 24 h, but the last two cases (ATL 5 and 7) did not show detectable levels of IL-6 even at 24 h. It is known that IL-6 mRNA is induced in monocytes and T-cells within 5 h and 24-48 h after culture initiation, respectively (Kishimoto, 1989). Considering the high purity of ATL cells used in the study (Table I) and early detection of IL-6 at 4 or 8 h of culture, the large amount of IL-6 detected in ATL-CMs seemed to be secreted by ATL cells themselves. However, normal T cells contaminated in the T cell preparations, if activated, could have contributed some of the IL 6 that was secreted into their cultures.

IL-6 promotes the growth of PHA-stimulated thymocytes and peripheral T-cells (Kishimoto, 1989). To examine whether the IL-6 enhances proliferation of ATL-cells, ATL from patients 1, 2, 5, 6 and 7 were incubated in the presence of IL-6 (20 ng $ml^{-1}$) or anti-IL-6 antibody (200 u $ml^{-1}$) for 72 h; proliferation was measured by $^3$H-thymidine ($^3$H-TdR) incorporation in the last 16 h of culture. The $^3$H-TdR uptake into ATL-cells was not influenced by either IL-6 or anti-IL-6 antibody (Table II).

Thus, we have demonstrated that ATL cells from four out of six patients secreted IL-6 spontaneously *in vitro* and that IL-6 was not detected in the sera of ATL patients in different phases of the disease. HTLV-I infection activates genes for

**Figure 1** IL-6 levels in conditioned media of ATL-cells. Cells ($1 \times 10^6$ $ml^{-1}$) were incubated for indicated periods of time. IL-6 levels in conditioned media were determined by ELISA in duplicates following the instructions of the manufacturer. The horseradish peroxidase-mediated colour reaction was measured by substracting absorbance at 650 nm (A. 650) from that at 492 nm (A. 492). The screened columns represent ranges of IL-6 levels in normal T-cell-CM (mean ± s.d., $n = 4$). The broken line show the lower limit of absorbance which can be converted to IL-6 concentration using standard curve. The closed circle shows IL-6 concentrations in complete medium used in cell culture. Values beside each point indicate calculated IL-6 concentrations (ng $ml^{-1}$).

Correspondence: H. Tsuda.
Received 1 March 1990; and in revised form 3 August 1990.

**Table I** Surface phenotypes of the mononuclear cells from ATL patients

| | % Positive cells[a] | | | | | | |
|---|---|---|---|---|---|---|---|
| *Patients* | *CD2* | *CD3* | *CD4* | *CD8* | *CD16* | *CD20* | *CD25* |
| ATL 1 | NT[b] | 42.3 | 85.6 | 5.7 | NT | NT | 20.4 |
| 2 | 99.9 | 99.2 | 99.6 | 7.7 | 0.2 | 0.0 | 78.1 |
| 3 | 99.6 | 96.4 | 91.9 | 5.4 | 4.1 | 0.2 | 88.6 |
| 4 | 98.1 | 51.9 | 96.4 | 1.9 | NT | 1.3 | 94.1 |
| 5 | 89.1 | 63.4 | 71.1 | 9.2 | NT | 9.4 | 45.2 |
| 6 | 97.7 | 74.6 | 88.5 | 6.3 | 2.7 | 0.4 | 73.7 |
| 7 | 99.0 | 29.7 | 98.6 | 0.8 | NT | 0.9 | 76.4 |

[a]The cells were stained by the direct or indirect immunofluorescence technique using monoclonal antibodies; OKT11, OKT3, OKT4, OKT8, OKNK, B1 or anti Tac, then analysed by flow cytometer (Cytoron, Ortho Diagnostic Systems, Tokyo, Japan) as described previously (Tsuda & Takatsuki, 1983, 1984). [b]not tested.

various cytokines and their receptors, but cytokine production is not solely regulated by the virus genome (Noma *et al.*, 1989). Multiple elements induced in T-cells by HTLV-I infection seem to contribute to the production of IL-6. The gene for IL-6 is located in chromosome 7p21 (Sehgel *et al.*, 1986). As the frequent chromosomal abnormalities of ATL are observed in chromosome 7 (Ueshima *et al.*, 1981), it is intriguing to speculate on the presence of a relationship between chromosome 7 abnormality and unregulated production of IL-6 presented in this study.

Despite *in vitro* overproduction of IL-6 by ATL-cells, we could not show detectable levels of IL-6 in the sera of ATL patients. Although the reason for this discrepancy is unclear, a more sensitive assay could show enhanced IL-6 levels in ATL sera.

$^{3}$H-TdR incorporation by ATL-cells was not affected by IL-6 or anti-IL-6 antibody added exogenously. Nevertheless, it is still possible that IL-6 may play an important role in the growth of ATL-cells *in vivo* since IL-6 reportedly induced IL-2 and IL-2 receptor expression in normal T-cells (Garman *et al.*, 1987; Tosato & Pike, 1988; Noma *et al.*, 1987). However, our preliminary data suggest that expression of IL-2 receptor on ATL cells was not apparently affected by either IL-6 or anti-IL-6 antibody (data not shown). In ATL, an autocrine mechanism through IL-2 and IL-2 receptor is still in debate (Tsuda & Takatsuki, 1983; Uchiyama *et al.*, 1985; Arima *et al.*, 1987). The clinical features that accompany ATL are diverse. Some of them may be explained by IL-6 production by ATL cells, such as the frequently observed elevation of C-reactive protein (CRP), accelerated erythrocyte sedimentation rate (ESR) and thrombocytosis (Castell *et al.*, 1989; Lotem *et al.*, 1989). Thus unregulated IL-6 production by ATL cells may be involved not only in pathogenesis but also in major clinical manifestations of the disease.

**Table II** Effect of IL-6 and anti-IL-6 antibody on proliferation of ATL-cells

| | $^{3}$H-Thymidine uptake (c.p.m. mean ± s.d.) | | |
|---|---|---|---|
| *Patients* | *None* | *IL-6 (20 ng ml$^{-1}$)* | *Anti-IL-6 (200 u ml$^{-1}$)*[a] |
| ATL 1 | 369 ± 24 | 361 ± 37 | 393 ± 29 |
| 2 | 401 ± 6 | 434 ± 41 | 416 ± 27 |
| 5 | 1880 ± 167 | 1653 ± 47 | NT[b] |
| 6 | 1231 ± 176 | 1291 ± 203 | 1317 ± 114 |
| 7 | 1559 ± 118 | 1485 ± 77 | NT |
| normal control | 1431 ± 126 | 1383 ± 123 | 1231 ± 17 |

[a]One unit (u) anti-IL-6 corresponds to titre to neutralise 1 u IL-6; [b]Not tested.

### References

ARIMA, N., DAITOKU, Y., YAMAMOTO, Y. & 7 others (1987). Heterogeneity in response to interleukin 2 and interleukin 2-producing ability of adult T-cell leukemia cells. *J. Immunol.*, **138**, 3069.

CASTELL, J.V., GOMEZ-LECHON, M.J., DAVID, M. & 3 others (1988). Recombinant human interleukin-6 (IL-6/BSF/HSF) regulates the synthesis of acute phase proteins in human hepatocytes. *FEBS Lett.*, **232**, 347.

GARMAN, R.D., JACOBS, K., CLARK, S.C. & RAULET, D.H. (1987). B-cell-stimulatory factor 2 (B2-interferon) functions as a second signal for interleukin-2 production by mature murine T-cells. *Proc. Natl Acad. Sci. USA*, **84**, 7629.

HACK, C.E., GROOT, E.D., FELT-BERMA, R.F. & 5 others (1989). Increased plasma levels of interleukin-6 in sepsis. *Blood*, **74**, 1704.

HIRANO, T., YASUKAWA, K., HARADA, H. & 4 others (1986). Complementary DNA for a novel human interleukin (BSF-2) that induces B lymphocytes to produce immunoglobulin. *Nature*, **324**, 73.

HORII, Y., MURAGUCHI, A., SUEMATSU, S. & 4 others (1988). Regulation of BSF-2/IL-6 production by human mononuclear cells: macrophage-dependant synthesis of BSF-2/IL-6 by T cells. *J. Immunol.*, **141**, 1529.

KAWANO, F., YAMAGUCHI, K., NISHIMURA, H., TSUDA, H. & TAKATSUKI, K. (1985). Variation in the clinical courses of adult T-cell leukemia. *Cancer*, **55**, 851.

KISHIMOTO, T. (1989). The biology of interleukin-6. *Blood*, **74**, 1.

KODAKA, T., UCHIYAMA, T., UMADOME, T.M. & UCHINO, H. (1989). Expression of cytokine mRNA in leukemic cells from adult T cell leukemia patients. *Jpn. J. Cancer Res.*, **80**, 50.

LOTEM, J., SHABO, Y. & SACHS, L. (1989). Regulation of megakaryocyte development by interleukin-6. *Blood*, **74**, 1545.

NOMA, T., MIZUTA, T., ROSEN, A., HIRANO, T., KISHIMOTO, T. & HONJO, T. (1987). Enhancement of the interleukin-2 receptor expression on T-cells by multiple B-lymphotropic lymphokines. *Immunol. Lett.*, **15**, 249.

NOMA, T., NAKAKUBO, H., SUGITA, M. & 4 others (1989). Expression of different combinations of interleukins by human T cell leukemic cell lines that are clonally related. *J. Exp. Med.*, **169**, 1853.

RAY, A., TATTER, S.B., SANTHANAM, U.M., HELFGOTT, D.C., MAY, L.G. & SEHGAL, P.B. (1989). Regulation of expression of interleukin-6. In *Regulation of the Acute Phase and Immune Responses: Interleukin-6*, Sehgal, P.B., Grienger, G. and Tosato, G. (eds) p. 353. New York Academy of Sciences: New York.

SEHGAL, P.B., ZILBERSTEIN, A., RUGGIERI, T. & 5 others (1986). Human chromosome 7 carries the B2 interferon gene. *Proc. Natl Acad. Sci. USA*, **83**, 5219.

TOSATO, G. & PIKE, S.E. (1988). Interferon-B2/interleukin-6 is a co-stimulant for human T lymphocytes. *J. Immunol.*, **141**, 1556.

TSUDA, H. & TAKATSUKI, K. (1983). Correlation of aberrant proliferation with T-cell growth factor in adult T-cell leukaemia cells. *Haematol. Oncol.*, **1**, 177.

TSUDA, H. & TAKATSUKI, K. (1984). Specific decrease in T3 antigen density in adult T-cell leukaemia cells I. Flow microfluorimetric analysis. *Br. J. Cancer*, **50**, 843.

UCHIYAMA, T., HORI, T., TSUDO, M. & 7 others (1985). Interleukin-2 receptor (Tac antigen) expressed on adult T-cell leukemia cells. *J. Clin. Invest.*, **76**, 446.

UCHIYAMA, T., KAMIO, M., KODAKA, T. & 5 others (1988). Leukemic cells from some adult T-cell leukemia patients proliferate in response to interleukin-4. *Blood*, **72**, 1182.

UESHIMA, Y., FUKUHARA, S., HATTORI, T., UCHIYAMA, T., TAKATSUKI, K. & UCHINO, H. (1981). Chromosomal studies in adult T-cell leukemia in Japan: Significance of trisomy 7. *Blood*, **58**, 420.